# Clinical Outcomes and Risk Factors for Surgical Failure Following Baerveldt Glaucoma Implant Surgery as a Primary Filtering Procedure

**DOI:** 10.3390/jcm15124649

**Published:** 2026-06-15

**Authors:** Kentaro Iwasaki, Ayami Katsuo, Shogo Arimura, Yoshihiro Takamura, Masaru Inatani

**Affiliations:** Department of Ophthalmology, Faculty of Medical Sciences, University of Fukui, Fukui 910-1193, Japan; kenkentaro0329@yahoo.co.jp (K.I.); a-tomoda@g.u-fukui.ac.jp (A.K.); leosunshine33@gmail.com (S.A.); ytakamura@hotmail.com (Y.T.)

**Keywords:** Baerveldt glaucoma implant, primary filtering procedure, clinical outcomes, risk factors

## Abstract

**Background/Objectives**: To evaluate the clinical outcomes and prognostic factors of Baerveldt glaucoma implant (BGI) surgery performed as a primary filtering procedure in eyes without prior glaucoma filtering surgery. **Methods**: This retrospective cohort study included 148 eyes of 148 patients who underwent BGI surgery with a 350-mm^2^ endplate at a single tertiary center. Surgical success was defined using three intraocular pressure (IOP)-based criteria: IOP > 21 mmHg (criterion A), >17 mmHg (criterion B), or >14 mmHg (criterion C), failure to achieve ≥ 20% IOP reduction, need for additional glaucoma surgery, loss of light perception, or persistent hypotony. Kaplan–Meier survival analysis and multivariable Cox proportional hazards regression were used to evaluate surgical outcomes and prognostic factors. **Results**: The 5-year cumulative probability of surgical success was 70.6%, 49.8%, and 27.6% for criteria A, B, and C, respectively. Mean IOP decreased significantly from 33.5 ± 10.0 mmHg preoperatively to 13.9 ± 4.0 mmHg at 5 years (*p* < 0.01); number of glaucoma medications decreased from 4.0 ± 1.2 to 1.8 ± 1.9 (*p* < 0.01). Younger age was associated with a higher risk of surgical failure (criterion A: hazard ratio [HR] 0.97, *p* < 0.01; criterion B: HR 0.98, *p* = 0.011; criterion C: HR 0.97, *p* < 0.01). More previous intraocular surgeries were associated with failure under criterion B (HR 1.30, *p* = 0.048). Early and late postoperative complications occurred in 34.5% and 14.2% of eyes, respectively; 20.9% required additional postoperative interventions. **Conclusions**: BGI surgery performed as a primary filtering procedure demonstrated favorable long-term IOP control in eyes without prior glaucoma filtering surgery. Younger age was identified as a consistent risk factor for surgical failure.

## 1. Introduction

Glaucoma is a leading cause of irreversible blindness worldwide, and surgical intervention is often required to achieve adequate intraocular pressure (IOP) control in advanced or medically uncontrolled cases. Trabeculectomy has long been considered the standard filtering procedure for lowering IOP; however, its surgical success is limited in eyes with conjunctival scarring, inflammation, or secondary glaucoma, where postoperative fibrosis can compromise filtration. In recent years, glaucoma drainage devices have been increasingly used as an effective alternative surgical option for the management of refractory glaucoma [1,2]. These include valved devices, such as the Ahmed glaucoma valve (AGV), and non-valved devices, such as the Baerveldt glaucoma implant (BGI), which differ in their flow characteristics and risk profiles [3]. The Tube Versus Trabeculectomy (TVT) Study demonstrated that tube-shunt surgery provides comparable or superior long-term outcomes compared with trabeculectomy in eyes with previous surgery [4]. Furthermore, the Primary Tube Versus Trabeculectomy (PTVT) Study extended these findings to patients without prior incisional glaucoma surgery, showing that tube-shunt surgery is a viable primary surgical option [5]. Compared with trabeculectomy, tube-shunt surgery is less dependent on conjunctival wound healing at the limbus and may provide more stable long-term IOP control in eyes at high risk for filtration failure. Several studies, including our previous reports, have reported favorable outcomes of BGI surgery in various glaucoma subtypes and in refractory cases [6,7,8,9]. However, most prior studies have focused on BGI implantation in eyes with a history of previous glaucoma surgery or in specific glaucoma subtypes. Although a previous report has suggested that primary BGI may be effective in neovascular glaucoma (NVG) [10], evidence regarding its outcomes as an initial filtering procedure remains limited, particularly in eyes without prior conventional filtering surgery and in real-world clinical settings.

Therefore, the present study aimed to evaluate the surgical outcomes of BGI implantation performed as a primary filtering procedure in eyes without prior glaucoma filtering surgery. In addition, we investigated prognostic factors associated with surgical failure to provide clinically relevant insights into surgical decision-making.

## 2. Materials and Methods

### 2.1. Patient Selection

The study protocol received approval from the Institutional Review Board of Fukui University Hospital and adhered to the principles outlined in the Declaration of Helsinki. Because of the retrospective nature of the study and the use of anonymized clinical data, the requirement for study-specific informed consent was waived, although all patients had provided consent for the surgical procedures themselves.

We retrospectively reviewed consecutive patients who underwent BGI surgery with a 350-mm^2^ endplate (BG101-350 or BG102-350; Abbott Medical Optics, Abbott Park, IL, USA) at Fukui University Hospital between April 2012 and March 2025. Eligible patients were aged ≥20 years and had glaucoma considered at high risk of failure with conventional filtering surgery, including secondary glaucoma, NVG, or eyes with conjunctival scarring due to prior ocular surgery. Eyes with no light perception, a history of prior glaucoma filtering procedures (including trabeculectomy, Ex-PRESS [Alcon Laboratories, Fort Worth, TX, USA], PreserFlo MicroShunt (Santen Pharmaceutical Co., Ltd., Osaka, Japan), BGI, or AGV), or insufficient postoperative follow-up (<6 months) were excluded. When both eyes of the same patient met the eligibility criteria, only the first-operated eye was included in the analysis. In this study, BGI was performed as the primary filtering procedure, and eyes with any history of prior filtering surgery were strictly excluded. Some patients included in the present study overlapped with those in our previous report [6]; however, the previous study evaluated overall BGI outcomes irrespective of surgical indication, whereas the present study specifically focused on BGI performed as a primary filtering procedure.

### 2.2. Surgical Procedures

The surgical technique for BGI implantation has been described previously [6]. In this study, a 350-mm^2^ endplate (BG101-350 or BG102-350) was used in all cases. When combined cataract surgery was indicated, phacoemulsification with intraocular lens implantation was performed through a 2.4-mm temporal clear corneal incision prior to BGI implantation. Following administration of subconjunctival and sub-Tenon’s anesthesia with xylocaine, a fornix-based conjunctival flap was created. The implant plate was inserted into the superotemporal quadrant beneath the rectus muscles and secured to the sclera approximately 10 mm posterior to the limbus. When significant conjunctival scarring was present in the superotemporal quadrant, an alternative quadrant was selected for plate placement. To prevent early postoperative hypotony, the tube was temporarily ligated using an 8-0 absorbable vicryl suture. After ligation with an 8-0 absorbable vicryl suture, tube occlusion was confirmed by irrigation, and the absence of fluid flow through the tube was used to verify complete sealing. Following creation of the tube entry site with a 23-gauge needle, the tube was positioned in either the anterior chamber or the ciliary sulcus. In vitrectomized eyes, pars plana tube placement into the vitreous cavity was performed when clinically appropriate. A 20- or 24-gauge microvitreoretinal lance was used to create the pars plana entry site, through which either a Hoffman elbow (BG102-350) or a straight tube (BG101-350) was inserted. The straight tube was trimmed to permit insertion of approximately 2–3 mm into the eye from the limbus. After insertion, tube length was reassessed intraoperatively and adjusted as necessary to avoid excessive intraocular tube length. Tube position was confirmed intraoperatively by direct visualization to ensure appropriate placement without contact with adjacent ocular structures. Sherwood slits were created in the tube with a 9-0 nylon needle to allow limited early aqueous flow before tube opening. The tube was then protected using either a half-thickness scleral flap or a preserved donor scleral patch graft. The scleral patch graft was secured using 9-0 nylon sutures. The Tenon’s capsule and conjunctiva were then closed together as a single layer using 8-0 absorbable vicryl sutures. In eyes with NVG, preoperative intravitreal anti–vascular endothelial growth factor (anti-VEGF) injections were administered when clinically indicated, based on the extent of iris or angle neovascularization, and the treating surgeon’s judgment. Following surgery, patients received a standardized postoperative medication regimen consisting of topical levofloxacin 1.5% or moxifloxacin 0.5% administered three times daily for 3 weeks, together with 0.1% betamethasone sodium phosphate three times daily for up to 6 months.

### 2.3. Data Collection

Clinical and demographic data were retrospectively extracted from medical records. Variables collected included patient age, sex, glaucoma subtype, best-corrected visual acuity (BCVA), preoperative and postoperative IOP, number of glaucoma medications, history of prior intraocular surgery, surgical details, postoperative complications, and additional postoperative interventions. For statistical analyses, visual acuity data were expressed as logarithm of the minimum angle of resolution (logMAR) values. In eyes with vision too poor for standard visual acuity measurement, the following decimal equivalents were used: counting fingers (0.00500), hand motions (0.00250), light perception (0.00125), and no light perception (0.00010). For the purpose of analysis, oral carbonic anhydrase inhibitors were considered equivalent to one topical glaucoma medication.

### 2.4. Primary Outcome Measure

The primary outcome of this study was surgical success, evaluated using three IOP-based criteria. These criteria were applied from 3 months postoperatively onward and were assessed irrespective of the use of glaucoma medications. Surgical failure was defined as insufficient IOP control, indicated by either failure to achieve at least a 20% reduction from the preoperative IOP or a postoperative IOP exceeding the following thresholds on two consecutive follow-up visits: >21 mmHg (criterion A), >17 mmHg (criterion B), or >14 mmHg (criterion C). IOP measurements obtained during routine clinical follow-up, typically conducted at intervals of up to 6 months, were used for this evaluation. Eyes were also considered to have failed if they required additional glaucoma surgery, including procedures involving removal of the BGI, or if they developed loss of light perception or persistent hypotony (defined as an IOP ≤ 5 mmHg). Eyes that did not meet any of these conditions were classified as surgical successes. Potential factors associated with surgical failure were further analyzed.

### 2.5. Secondary Outcome Measures

Secondary endpoints comprised changes in IOP and the number of glaucoma medications over time, as well as the incidence of early and late postoperative complications, visual acuity outcomes, and the need for additional postoperative interventions.

### 2.6. Statistical Analysis

Longitudinal changes in postoperative IOP and the number of glaucoma medications were evaluated using linear mixed-effects models with time as a fixed effect and patient as a random effect. Changes in visual acuity from baseline to the final follow-up visit were analyzed using the Wilcoxon signed-rank test. Time-to-failure outcomes were assessed using Kaplan–Meier survival analysis, and differences between groups were evaluated using the log-rank test. To identify factors associated with surgical failure, multivariable Cox proportional hazards regression models were applied. Covariates were selected a priori based on clinical relevance and findings from previous studies. Statistical analyses were performed with JMP Pro version 17.2.0 (SAS Institute Inc., Cary, NC, USA). A two-tailed *p*-value < 0.05 was considered indicative of statistical significance.

## 3. Results

### 3.1. Patient Characteristics

A total of 148 eyes from 148 patients were included in the analysis. Baseline characteristics are summarized in Table 1. All patients were Japanese. The mean follow-up duration was 55.7 ± 37.8 months. Combined cataract surgery was performed in 17 eyes (11.5%); all phakic eyes underwent simultaneous cataract extraction at the time of BGI implantation. No cases involved combined vitrectomy. Among the 70 eyes with NVG, 35 eyes (50.0%) received an intravitreal anti-VEGF injection within 1 month before BGI implantation. Age at surgery and baseline and final IOP values according to glaucoma subtype are summarized in Supplementary Table S1.

### 3.2. Primary Outcome Measure

**Figure 1:**
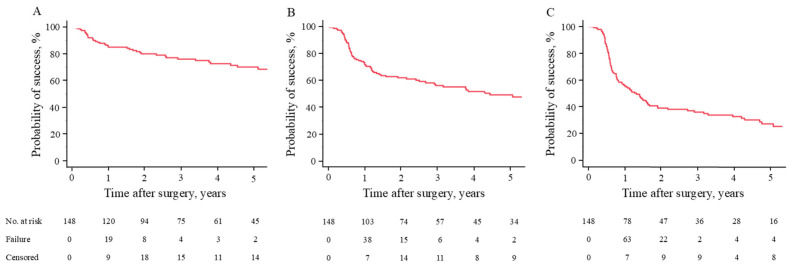
Kaplan–Meier survival curves showing surgical success according to three IOP-based failure criteria. Criterion (**A**) was defined as IOP > 21 mmHg, <20% reduction from baseline, requirement for additional glaucoma surgery, loss of light perception, or hypotony ≤ 5 mmHg. Criteria (**B**,**C**) were defined similarly, with IOP thresholds of >17 mmHg and >14 mmHg, respectively. IOP, intraocular pressure.

Kaplan–Meier survival curves for surgical success according to failure criteria A, B, and C are presented in Figure 1. The cumulative probability of surgical success at 1, 3, and 5 years was 85.5%, 76.6%, and 70.6% for criterion A; 72.4%, 56.7%, and 49.8% for criterion B; and 55.8%, 36.4%, and 27.6% for criterion C, respectively.

Table 2 summarizes the reasons for surgical failure according to criterion A. Overall, 42 eyes (28.4%) were classified as failures. The most common cause of failure was inadequate IOP reduction (22 eyes, 14.9%). Among these, nine eyes subsequently required additional glaucoma surgery. In total, 18 eyes underwent further surgical intervention for glaucoma during the follow-up period, including micropulse transscleral cyclophotocoagulation in 15 eyes, AGV implantation in two eyes, and trabeculectomy in one eye. The choice of secondary surgical procedure depended on the severity of IOP elevation, visual potential, conjunctival condition, associated ocular comorbidities, and patient preference. In many cases, less invasive procedures such as micropulse transscleral cyclophotocoagulation were preferred because of extensive conjunctival scarring or complex ocular conditions.

### 3.3. Multivariable Analysis of Prognostic Factors for Surgical Failure

Potential prognostic factors for surgical failure, including age, glaucoma type, preoperative IOP, number of preoperative glaucoma medications, combined cataract surgery, number of previous intraocular surgeries, and preoperative visual acuity, were evaluated using multivariable Cox proportional hazards regression models (Table 3). Younger age was consistently associated with a higher risk of surgical failure across all criteria (criterion A: hazard ratio [HR] 0.97, *p* < 0.01; criterion B: HR 0.98, *p* = 0.011; criterion C: HR 0.97, *p* < 0.01). In addition, a greater number of previous intraocular surgeries was significantly associated with an increased risk of failure under criterion B (HR 1.30, *p* = 0.048). No other variables were identified as significant predictors.

**Figure 2:**
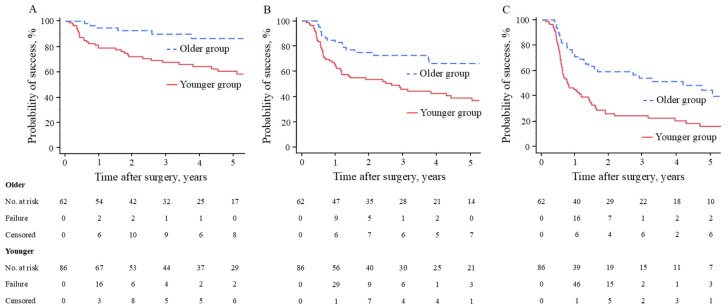
Kaplan–Meier survival curves comparing surgical success between older (≥70 years) and younger (<70 years) patients according to three IOP-based failure criteria. Criterion (**A**) was defined as IOP > 21 mmHg, <20% reduction from baseline, requirement for additional glaucoma surgery, loss of light perception, or hypotony ≤ 5 mmHg. Criteria (**B**,**C**) were defined similarly, with IOP thresholds of >17 mmHg and >14 mmHg, respectively. IOP, intraocular pressure.

To further illustrate the impact of age on surgical outcomes, a supplementary Kaplan–Meier analysis stratified by age (≥70 vs. <70 years) was performed. Figure 2 presents Kaplan–Meier survival curves comparing surgical outcomes between older (≥70 years) and younger (<70 years) patients based on failure criteria A, B, and C. Across all criteria, the probability of surgical success was significantly higher in the older group than in the younger group (log-rank test, *p* < 0.01 for all comparisons). For criterion A, the cumulative probabilities of success at 1, 3, and 5 years were 94.8%, 89.9%, and 86.4% in the older group and 78.9%, 67.6%, and 60.7% in the younger group, respectively. For criterion B, the corresponding values were 83.1%, 72.8%, and 66.5% versus 65.0%, 46.2%, and 39.3%, and for criterion C, 71.0%, 54.1%, and 44.7% versus 45.3%, 24.4%, and 16.1%, respectively.

### 3.4. Secondary Outcome Measures

Changes in IOP and the number of glaucoma medications over time are summarized in Table 4. In eyes requiring additional glaucoma surgery, observations recorded after the reoperation were excluded from the longitudinal outcome analyses. Linear mixed-effects modeling demonstrated significant longitudinal reductions in both postoperative IOP and the number of glaucoma medications over time (both *p* < 0.01). Mean IOP decreased significantly from 33.5 ± 10.0 mmHg preoperatively to 13.9 ± 4.0 mmHg at 5 years. Similarly, the mean number of glaucoma medications decreased from 4.0 ± 1.2 to 1.8 ± 1.9.

Table 5 summarizes postoperative complications. A total of 79 early postoperative complication events occurring within 3 months after surgery were observed in 51 eyes (34.5%). The most frequent early complication was hyphema (32 eyes, 21.6%), which resolved spontaneously in most cases (29 eyes). One eye required intravitreal anti-VEGF injection, and two eyes required anterior chamber washout. Hemorrhagic complications occurred more frequently in eyes with NVG; among the 32 eyes with postoperative hyphema, 16 eyes (50.0%) had NVG. In addition, NVG accounted for 13 of 18 eyes (72.2%) with early vitreous hemorrhage. Late postoperative complications (>3 months) were observed in 27 events involving 21 eyes (14.2%). The most common late complication was vitreous hemorrhage (7 eyes, 4.7%), all of which occurred in eyes with NVG. Six of these eyes required pars plana vitrectomy, while one eye was treated with intravitreal anti-VEGF injection. Among the five eyes that developed bullous keratopathy, two had exfoliation glaucoma and three had secondary glaucoma. All eyes had tube insertion into the anterior chamber. Preoperative corneal endothelial cell density was below 1000 cells/mm^2^ in three eyes, unavailable in one eye, and 2387 cells/mm^2^ in one eye. In the latter case, persistent postoperative hypotony with a shallow anterior chamber was considered to have contributed to subsequent endothelial decompensation.

Postoperative interventions are summarized in Table 6. A total of 41 intervention events were performed in 31 eyes (20.9%). The most frequent intervention was pars plana vitrectomy (13 eyes, 8.9%). Pars plana vitrectomy was performed for the management of vitreous hemorrhage, retinal detachment, intraocular lens dislocation, endophthalmitis, or silicone oil removal. Intravitreal anti-VEGF injections were administered for the treatment of hyphema or vitreous hemorrhage in eyes with NVG, as well as for macular edema secondary to diabetic retinopathy or retinal vein occlusion. Intravitreal or sub-Tenon’s triamcinolone acetonide injections were performed to treat macular edema associated with diabetic retinopathy.

Visual acuity (logMAR) significantly worsened from 0.95 ± 0.83 preoperatively to 1.20 ± 0.99 at the final follow-up (*p* < 0.01). At the final visit, visual acuity had worsened in 81 eyes (54.7%), improved in 40 eyes (27.0%), and remained unchanged in 27 eyes (18.3%). Among the eyes with deterioration, five eyes (3.4%) progressed to no light perception. Three of these eyes had NVG associated with proliferative diabetic retinopathy, one had exfoliation glaucoma, and one had secondary glaucoma. Preoperative visual acuity was already severely impaired in all eyes, ranging from hand motions to 0.04. In three eyes, postoperative IOP was adequately controlled; however, visual loss progressed because of advanced underlying ocular disease. One eye with secondary glaucoma had persistent IOP elevation but did not undergo additional surgery because of limited visual potential and patient preference, whereas the remaining eye with NVG showed uncontrolled IOP despite treatment.

## 4. Discussion

In this study, we evaluated the clinical outcomes of BGI surgery performed as a primary filtering procedure in eyes without prior glaucoma filtering surgery. Our results demonstrated favorable long-term IOP control, with a 5-year success rate of 70.6% under the least stringent criterion. In addition, we identified younger age as a consistent risk factor for surgical failure across all IOP-based criteria.

The role of tube-shunt surgery as an initial surgical option has been increasingly recognized. The PTVT Study demonstrated that tube-shunt surgery can achieve outcomes comparable to trabeculectomy in eyes without prior incisional glaucoma surgery [5]. In the PTVT Study, the 5-year success rates in the tube group were reported as 58%, 51%, and 36% for criteria A, B, and C, respectively. Our findings further support this concept by specifically evaluating BGI as a primary filtering procedure in a real-world cohort. In the present study, the 5-year success rates were 70.6%, 49.8%, and 27.6% for criteria A, B, and C, respectively. While the success rate for criterion A was higher in our cohort, the rates for stricter criteria (B and C) were comparable or slightly lower than those reported in the PTVT Study. These differences may be attributable to variations in patient characteristics, particularly the higher proportion of neovascular and secondary glaucoma in our study population, in which trabeculectomy outcomes are often suboptimal. In such cases, tube-shunt surgery may provide more stable long-term IOP control due to its reduced dependence on subconjunctival wound healing at the limbus. In addition, our study reflects real-world clinical practice, which may better capture outcomes in more complex and heterogeneous patient populations.

A key finding of this study was that younger age was strongly associated with an increased risk of surgical failure. In the multivariable analysis, age was evaluated as a continuous variable, and this association was consistently observed across all IOP criteria. For illustrative purposes, Kaplan–Meier survival curves were additionally generated using an age cutoff of 70 years, demonstrating lower surgical success rates in younger patients (Figure 2). One possible explanation is that younger patients may exhibit a more pronounced fibrotic response around the endplate, leading to increased resistance to aqueous outflow. In contrast, reduced wound healing responses in older patients may contribute to more favorable long-term outcomes. Similar trends have been reported in previous studies of tube-shunt surgery [6,8,11,12], suggesting that age-related differences in tissue response play an important role in surgical success [13]. In addition, one report has suggested that age-related impairments in wound healing are associated with fibroblast dysfunction [14]. However, younger patients in our cohort also more frequently had NVG and other secondary glaucomas. Therefore, the observed association between younger age and surgical failure may partly reflect differences in underlying disease characteristics and systemic comorbidities rather than age-related wound-healing responses alone. These findings suggest that younger patients may remain at relatively high risk of surgical failure even after BGI implantation. Therefore, careful long-term follow-up and individualized surgical planning may be particularly important in this population. Optimization of postoperative wound healing through meticulous control of inflammation, intensive postoperative corticosteroid therapy, and early recognition of excessive encapsulation may help reduce fibrotic dysfunction. In younger eyes with aggressive wound healing responses, alternative surgical strategies or earlier consideration of adjunctive procedures may also be warranted; however, further studies are needed to determine the optimal surgical approach for these patients.

An increased number of prior intraocular surgeries was associated with a higher risk of surgical failure under criterion B in this study, although this relationship was not observed consistently across all criteria. This finding suggests that the influence of prior surgical history may be modest and dependent on the definition of success. One possible mechanism is that repeated intraocular procedures may induce cumulative changes in the subconjunctival tissue, including enhanced fibrotic remodeling, which could reduce the efficiency of aqueous humor drainage [13]. A previous report has shown that eyes with poor bleb function demonstrate increased extracellular matrix deposition and heightened fibroblast activity following tube-shunt surgery [15]. In addition, surgical manipulation of the conjunctiva itself may further augment fibroblast activation [16,17]. Furthermore, several studies have suggested that a greater number of prior intraocular surgeries is associated with an increased risk of surgical failure [6,7,9,12]. Taken together, these observations suggest that eyes with multiple prior intraocular surgeries may be less likely to achieve lower IOP targets after BGI implantation. However, the inconsistent association observed across the three criteria may partly reflect differences in statistical power and model stability, particularly under criterion A, where the number of failure events was relatively limited. Interestingly, glaucoma subtype, including NVG, was not identified as a significant risk factor for surgical failure in the multivariable analysis. This finding may reflect the mechanism of tube-shunt surgery, which bypasses the trabecular outflow pathway and is less affected by anterior segment pathology and conjunctival scarring compared with trabeculectomy. Although primary BGI has been reported to be effective in NVG, evidence remains limited. Our results suggest that BGI may provide stable outcomes across a broad range of glaucoma subtypes, even in high-risk eyes.

Regarding postoperative complications, early complications occurred in 19% and late complications in 22% of patients in the tube group of the PTVT Study [18]. In contrast, our study showed a higher rate of early complications (34.5%) but a lower rate of late complications (14.2%). The higher incidence of early complications in our cohort may be attributed to the increased frequency of hemorrhagic events, such as hyphema and vitreous hemorrhage, likely reflecting the higher proportion of NVG included in this study population. Hemorrhagic complications remain an important concern following BGI implantation, particularly in eyes with NVG. Several strategies may help reduce the risk of postoperative bleeding, including preoperative intravitreal anti-VEGF injections to promote regression of iris and angle neovascularization, optimization of systemic conditions such as diabetes mellitus and hypertension, and meticulous intraoperative hemostasis. In addition, our findings suggested that lower postoperative day 1 IOP and tube insertion into the anterior chamber or ciliary sulcus were associated with an increased risk of hyphema [19]. Therefore, careful measures to avoid excessive early postoperative hypotony and consideration of vitreous cavity tube insertion when clinically appropriate may help reduce the risk of hemorrhagic complications. Despite these differences, most complications in the present study were transient and manageable and did not preclude favorable long-term IOP control.

Visual acuity worsened considerably over the follow-up period. However, this finding should be interpreted with caution, as the study population included a large proportion of eyes with advanced disease and NVG. Visual prognosis is generally poor in NVG, even with appropriate medical and surgical management [20,21,22]. In addition, postoperative complications such as vitreous hemorrhage and progression of underlying retinal disease may have contributed to visual decline, rather than the surgical procedure itself.

This study has some limitations that should be considered when interpreting the results. First, the retrospective design may have introduced selection bias, and both surgical indications and postoperative management were not standardized. In particular, the use of glaucoma medications and follow-up strategies were determined at the discretion of the treating surgeons, which may have influenced the observed surgical outcomes. Second, this was a single-center study conducted in a Japanese population, potentially limiting the generalizability of the findings to other clinical settings or ethnic groups. Third, the lack of a control group, such as trabeculectomy, prevents direct comparison of surgical efficacy between different procedures. In addition, some clinically relevant data, including detailed optic nerve findings, visual field parameters, and corneal endothelial cell density, were not consistently available, which may have limited the comprehensive assessment of surgical outcomes and complications. Moreover, tube insertion site was not analyzed as an independent prognostic factor because it was closely associated with prior vitrectomy status and underlying ocular characteristics, particularly posterior segment disease, which may have introduced substantial confounding. Finally, although multivariable analyses were performed to adjust for potential confounders, residual confounding due to unmeasured variables cannot be excluded. Further prospective studies with standardized protocols and more comprehensive data collection are warranted to validate these findings.

From a clinical perspective, the present findings may help refine patient selection and postoperative management for primary BGI surgery. Younger patients and those with multiple prior intraocular surgeries may warrant closer long-term follow-up because of their potentially higher risk of surgical failure. In addition, recognition of these risk factors may facilitate more individualized surgical planning and patient counseling before surgery. Future studies are needed to determine whether risk-stratified management strategies can further improve long-term outcomes following BGI implantation.

## 5. Conclusions

BGI surgery demonstrated favorable long-term IOP control in eyes without prior glaucoma filtering surgery, particularly in patients at high risk for failure with conventional filtering procedures. Younger age was identified as a consistent risk factor for surgical failure. These findings may help guide surgical decision-making in the management of complex glaucoma cases.

## Figures and Tables

**Table 1:** Baseline characteristics of the study population.

Characteristic	Total (*n* = 148)
Age (years), mean ± SD	65.8 ± 13.5
Sex, *n* (%)	
Male	96 (64.9)
Female	52 (35.1)
Diabetes mellitus, *n* (%)	83 (56.1)
Hypertension, *n* (%)	71 (48.0)
Visual acuity (logMAR), mean ± SD	0.95 ± 0.83
IOP (mmHg), mean ± SD	33.5 ± 10.0
Glaucoma medications, n, mean ± SD	4.0 ± 1.2
Previous intraocular surgeries, n, mean ± SD	1.9 ± 1.0
Previous trabeculotomy, *n* (%)	11 (7.4)
Previous pars plana vitrectomy, *n* (%)	80 (54.1)
Axial length (mm), mean ± SD	24.9 ± 2.3
Type of glaucoma, *n* (%)	
Primary open-angle glaucoma	22 (14.9)
Primary angle-closure glaucoma	5 (3.3)
Exfoliation glaucoma	22 (14.9)
Neovascular glaucoma	70 (47.3)
Other secondary glaucoma	29 (19.6)
Lens status, *n* (%)	
Pseudophakia	126 (85.1)
Aphakia	5 (3.4)
Phakia combined with cataract surgery	17 (11.5)
Type of implant, *n* (%)	
BG 101-350	104 (70.3)
BG 102-350	44 (29.7)
Tube insertion position, *n* (%)	
Anterior chamber	47 (31.8)
Ciliary sulcus	39 (26.3)
Pars plana	62 (41.9)
Endplate position, *n* (%)	
Superotemporal	145 (98.0)
Superonasal	2 (1.3)
Inferonasal	1 (0.7)

IOP, intraocular pressure; logMAR, logarithm of minimum angle of resolution; SD, standard deviation.

**Table 2:** Reasons for surgical failure according to criterion A.

Reason for Surgical Failure	Total ^a^ (*n* = 42)
Inadequate IOP reduction ^b,c^	22 (14.9)
Reoperation for glaucoma	9 (6.1)
Hypotony ^d^	5 (3.4)
Removal of tube shunt	3 (2.0)
Loss of light perception	3 (2.0)

^a^ Data are presented as the number of eyes (%). ^b^ IOP > 21 mmHg or <20% reduction from baseline IOP on two consecutive follow-up visits after 3 months. ^c^ Some eyes required additional glaucoma surgery following failure due to inadequate IOP reduction. ^d^ IOP ≤ 5 mmHg on two consecutive follow-up visits after 3 months.

**Table 3:** Multivariable analysis to identify prognostic risk factors for failure using Cox proportional hazards regression models.

	Criterion					
	A		B		C	
	HR (95% CI)	*p*-Value	HR (95% CI)	*p*-Value	HR (95% CI)	*p*-Value
Age (per year)	0.97 (0.95–0.99)	<0.01	0.98 (0.96–0.99)	0.011	0.97 (0.96–0.99)	<0.01
Type of glaucoma (NVG/other)	1.25 (0.67–2.36)	0.48	1.21 (0.75–1.94)	0.43	1.15 (0.76–1.72)	0.51
Preoperative IOP (per mmHg)	1.01 (0.97–1.04)	0.75	1.01 (0.99–1.04)	0.31	1.02 (0.99–1.04)	0.085
Preoperative glaucoma medication (per medication)	1.15 (0.89–1.51)	0.30	1.12 (0.93–1.38)	0.25	1.64 (0.66–4.32)	0.29
Combined surgery (alone/combined cataract surgery)	0.94 (0.34–3.34)	0.91	0.67 (0.32–1.54)	0.33	0.60 (0.32–1.19)	0.14
Number of previous intraocular surgeries (per surgery)	1.23 (0.90–1.63)	0.19	1.30 (1.00–1.65)	0.048	1.09 (0.86–1.35)	0.46
Preoperative visual acuity (per 1.0 logMAR)	0.79 (0.53–1.16)	0.24	1.02 (0.76–1.35)	0.89	1.00 (0.77–1.30)	0.99

CI, confidence interval; HR, hazard ratio; NVG, neovascular glaucoma.

**Table 4:** Longitudinal changes in intraocular pressure and glaucoma medication use.

Time Point	IOP (mmHg)	Number of Medications	Number of Eyes
Preoperative	33.5 ± 10.0	4.0 ± 1.2	148
1 month	19.9 ± 8.6	1.9 ± 2.1	148
3 months	16.0 ± 7.0	1.9 ± 1.9	146
6 months	14.0 ± 4.9	1.9 ± 1.9	140
1 year	13.7 ± 4.2	1.9 ± 1.9	132
2 years	13.9 ± 3.8	2.0 ± 2.0	110
3 years	13.9 ± 4.1	2.0 ± 1.8	88
4 years	14.0 ± 4.0	1.8 ± 1.8	75
5 years	13.9 ± 4.0	1.8 ± 1.9	61

Data are presented as the mean ± standard deviation.

**Table 5:** Early and late postoperative complications.

Total ^a^ (*n* = 148)	Early ^b^	Late ^c^
Hyphema	32 (21.6)	0 (0.0)
Shallow or flat anterior chamber	6 (4.1)	1 (0.7)
Wound dehiscence	1 (0.7)	0 (0.0)
Choroidal detachment	19 (12.8)	0 (0.0)
Vitreous hemorrhage	18 (12.2)	7 (4.7)
Retinal detachment	1 (0.7)	0 (0.0)
IOL dislocation	1 (0.7)	1 (0.7)
Tube erosion	0 (0.0)	2 (1.4)
Removal of tube shunt	1 (0.7)	5 (3.4)
Endophthalmitis	0 (0.0)	1 (0.7)
Bullous keratopathy	0 (0.0)	5 (3.4)
Persistent diplopia	0 (0.0)	3 (2.0)
Corneal infection	0 (0.0)	2 (1.4)
Total number of eyes with postoperative complications ^d^	51 (34.5)	21 (14.2)

^a^ Data are presented as the number of eyes (%) calculated from the total study population (*n* = 148). ^b^ Early postoperative complications that occurred within 3 months after surgery. ^c^ Late postoperative complications that occurred more than 3 months after surgery. ^d^ Some eyes had more than one complication. IOL, intraocular lens.

**Table 6:** Postoperative interventions.

Intervention	Total ^a^ (*n* = 41)
Anterior chamber reformation	6 (4.1)
Anterior chamber washing	2 (1.4)
Intravitreal injection of anti-VEGF	13 (8.9)
Intravitreal injection of triamcinolone acetonide	2 (1.4)
Sub-Tenon’s injection of triamcinolone acetonide	1 (0.7)
Additional suture of wound	1 (0.7)
Pars plana vitrectomy	13 (8.9)
Penetrating keratoplasty	1 (0.7)
Tube revision with patch graft	2 (1.4)
Total number of eyes with postoperative interventions ^b^	31 (20.9)

^a^ Data are presented as the number of eyes (%) calculated from the total study population (*n* = 148). ^b^ Some eyes had more than one intervention. VEGF, vascular endothelial growth factor.

## Data Availability

The original contributions presented in this study are included in the article and its Supplementary Materials. Further inquiries can be directed to the corresponding author.

## Supplementary Materials

The following supporting information can be downloaded at: https://www.mdpi.com/article/10.3390/jcm15124649/s1, Supplementary Table S1: Age at surgery and baseline and final IOP according to glaucoma subtype.

## Abbreviations

The following abbreviations are used in this manuscript:

AGV	Ahmed glaucoma valve
anti-VEGF	anti–vascular endothelial growth factor
BCVA	best-corrected visual acuity
BGI	Baerveldt glaucoma implant
CI	confidence interval
HR	hazard ratio
IOL	intraocular lens
IOP	intraocular pressure
logMAR	logarithm of the minimal angle of resolution
NVG	neovascular glaucoma
PTVT	Primary Tube Versus Trabeculectomy
SD	standard deviation
TVT	Tube Versus Trabeculectomy
