# Supplementary material for: Clinical Outcomes and Risk Factors for Surgical Failure Following Baerveldt Glaucoma Implant Surgery as a Primary Filtering Procedure
Source: J Clin Med. 2026 Jun 15;15(12):4649. doi: 10.3390/jcm15124649 (PMC13301798; doi:10.3390/jcm15124649)
Supplement: Supplementary file 1 [file jcm-15-04649-s001.zip › jcm-4323624-supplementary.pdf]

Supplementary Information

Clinical Outcomes and Risk Factors for Surgical Failure Following Baerveldt Glaucoma Implant Surgery as a Primary Filtering Procedure

Kentaro Iwasaki[1], Ayami Katsuo[1], Shogo Arimura[1], Yoshihiro Takamura[1], Masaru Inatani[1]*

[1] Department of Ophthalmology, Faculty of Medical Sciences, University of Fukui, Fukui 910-1193, Japan

***Corresponding author**

Masaru Inatani

23-3 Shimoaizuki, Matsuoka, Eiheiji, Yoshida, Fukui 910-1193, Japan

Phone number: +81-776-61-8400

Fax number: +81-776-61-8131

Email address: inatani@u-fukui.ac.jp

Supplementary Table S1. Age at surgery and baseline and final IOP according to glaucoma subtype.

Type of glaucoma	Age at surgery (years)	Baseline IOP (mmHg)	Final IOP (mmHg)
Primary open-angle glaucoma	68.1 ± 12.6	28.0 ± 8.8	14.4 ± 4.6
Primary angle-closure glaucoma	77.0 ± 8.7	39.4 ± 13.0	11.0 ± 4.2
Exfoliation glaucoma	77.4 ± 8.8	32.0 ± 11.0	11.5 ± 4.5
Neovascular glaucoma	63.6 ± 11.7	33.5 ± 9.3	15.2 ± 6.6
Other secondary glaucoma	58.6 ± 15.2	37.7 ± 9.6	16.2 ± 9.4

Data are presented as mean ± standard deviation (SD).
